# Factors affecting response and resistance to venetoclax in acute myeloid leukemia

**DOI:** 10.3389/fonc.2025.1577908

**Published:** 2025-09-15

**Authors:** Michael D. Diamantidis

**Affiliations:** Thalassemia and Sickle Cell Disease Unit, Department of Hematology, General Hospital of Larissa, Larissa, Greece

**Keywords:** acute myeloid leukemia (AML), response, resistance, venetoclax (VEN), BCL2 (B-cell lymphoma 2) inhibition, azacitidine (AZA), hypomethylating agents (HMAs), MCL1 (myeloid cell leukemia sequence 1) overexpression

## Abstract

The use of the BCL2 inhibitor venetoclax in combination with hypomethylating agents (HMA) is a revolution for the treatment of frail and elderly acute myeloid leukemia (AML) patients. This effective treatment strategy is increasingly more and more applicable for other subsets of AML patients and is currently being tested in numerous clinical trials in combination with other drugs in all treatment lines. In particular, venetoclax combinations can also serve as a definitive therapy or as an effective bridge to allogeneic hematopoietic stem cell transplantation (HSCT). However, the factors affecting response to venetoclax in the abovementioned AML patients are not completely clear and understood until today. The aim of this review is to describe the molecular and clinical patterns of response and durable remission of venetoclax-based combinations in AML patients. Hence, mutations in IDH1, IDH2, ASXL1, NPM1, DDX41, chromatin-cohesin complex and splicing-factor genes predict superior response to venetoclax, while inferior response to the drug has been observed for FLT3-ITD, KRAS, NRAS and TP53 gene mutations. Intriguingly, the achievement of measurable residual disease (MRD) negativity in the first four cycles of venetoclax administration characterizes a subgroup of NPM1-mutated AML patients with a more favorable outcome. Even though focus will be given on factors influencing response to the drug in this review, the main mechanisms of resistance to venetoclax in AML patients will also be discussed.

## Introduction

The combination therapy of venetoclax and azacitidine (VEN-AZA) has demonstrated substantial benefits for patients with acute myeloid leukemia (AML) who are ineligible for intensive chemotherapy, particularly for those with specific molecular characteristics, such as NPM1-mutated AML or IDH-mutated AML (1). This regimen [Venetoclax 400 mg per os; days 1-28) and azacitidine (75 mg/m^2^; subcutaneously; days 1-7/28-day cycle] became the cornerstone and standard of care for the elderly and frail patients with AML, irrespective of the mutational status, based on the results of the VIALE-A randomized clinical trial (2). The VEN-AZA combination was associated with a statistically significant longer median overall survival (OS) of 14.7 months versus only 9.6 months for those who had received AZA alone (2). The latest 3-year follow-up of the study was also in favor of the combination, showing a three-year survival rate of 25% for VEN-AZA versus 10% for AZA alone (3).

However, the combination VEN-AZA often exhibits limited durability in response for numerous patients. In fact, response patterns to VEN remain poorly understood. Certain patients with AML demonstrate a remarkable response to the initial cycles of the drug, whereas others display various levels of response or no response at all. Consequently, research has increasingly concentrated on how more complex regimens may enhance outcomes, particularly through the incorporation of targeted drugs, such as FLT-3 or IDH inhibitors to VEN-AZA.

Moreover, despite the introduction of innovative techniques such as BH3 profiling to stratify AML patients undergoing VEN treatment in clinical trial settings (4), there is an absence of clinically relevant biomarkers, that predict the response to the combination of VEN-AZA. Conversely, resistance to the treatment is observed with the emergence of novel mutations compared to the initial ones. Despite the association of anti-apoptotic BCL2 family proteins with AML pathogenesis, the specific functional significance of individual proteins, including BCL2, BCL-xL, MCL1, BAX, and BFL1, remains inadequately clarified. The expression of these proteins is altered during the progression of AML by the administration of the selective BCL2 inhibitor VEN (5–8). The latter offers insights into the mechanisms of resistance to VEN in AML, suggesting potential treatment targets.

This review aims to elucidate the complex mechanisms of response and resistance to VEN in AML patients unfit for intensive therapy.

## Response to venetoclax

The use of BCL2 inhibitors, like VEN, in combination with hypomethylating agents (HMAs) has been associated with increased overall survival (OS) in AML patients over 65 years, who have BCL2 overexpression (9). The observed synergy between AZA and VEN can be attributed to various mechanisms. AZA induces pro-apoptotic modifications, including a reduction in MCL1 protein level, thereby facilitating the apoptotic properties of VEN (10, 11). Furthermore, through an increase in NOXA levels, AZA primes leukemic cells to VEN-induced apoptosis (12, 13). In addition, HMA and VEN combinations induce reactive oxygen species (ROS) accumulation and mitochondrial ROS production in leukemic cells, triggering the apoptotic mechanisms and facilitating the oxidative death of AML cells (14–16).

An intriguing observation was that AML patients with IDH mutations (either IDH1 or IDH2) showed high remission rates with an OS of 24.5 months and a composite complete remission CRc (CR + CRi) rate of 79% when treated with VEN-AZA (17). These percentages were much higher compared to the respective data of the VIALE-A study, which initiated VEN plus HMAs in frail or elderly AML patients ineligible for intensive chemotherapy (median OS: 14.7 months, CR/CRi: 66.4%) (2). Despite the small sample size in the molecular subgroups, the limited number of patients in the AZA group and the lack of thorough analysis between the IDH-mutant isoforms and concomitant mutations, the combination of VEN-AZA emerged as a very good competitive candidate for the IDH-mutated AML, apart from treatment option with the IDH inhibitors [enasidenib, ivosidenib (IVO), olutasidenib] (17, 18). Hence, one of the major factors predicting a good response to VEN-AZA in AML patients is the presence of IDH mutations (3, 18–20).

Intriguingly, among the IDH2-mutated subgroup a CRc rate of 86%, a CR rate of 56% and a not reached median OS have been observed, demonstrating a unique benefit for these AML patients with VEN-AZA. In addition, patients harboring the IDH1 mutation and receiving the combination of VEN-AZA also show a favorable response (CRc: 67%, CR: 27%, median OS: 15 months) (17). The latter is superior to the IDH1 inhibitor IVO in the first line treatment of AML, as the IVO data indicate a CRc rate of 42% and an OS of 12.6 months (21). Nevertheless, the approved combination of IVO plus AZA in IDH1-mutated AML demonstrates a median OS of 24 months, an event-free survival (EFS) of 37% at 12 months, a CRc rate of 53%, along with a median duration of response of 22.1 months (22). AML patients have nowadays many treatment options with the approval of novel targeted therapies, except for the combination of VEN-AZA. The most important clinical trials involving VEN, AZA and their combinations in AML patients are shown in **Table 1**. Regarding toxicity, grade ≥3 is provided, unless otherwise stated for any grade side effects.

**Table 1:** Clinical trials involving venetoclax, azacitidine, decitabine, ivosidenib, gilteritinib, quizartinib, cladribine and their combinations in patients with acute myeloid leukemia (AML) ineligible for intensive chemotherapy.

Reference	Trial phase	Title of the trial	Targeted drugs – dose	Study population	Response rates	Grade 3–4 toxicity	Study status	Clinical trial identifier
DiNardo CD et al, N Engl J Med, 2020 (2)	Phase III Interventional Randomized	A Study of Venetoclax in Combination With Azacitidine Versus Azacitidine in Treatment Naïve Participants With Acute Myeloid Leukemia Who Are Ineligible for Standard Induction Therapy (VIALE-A)	**VEN:** target dose 400 mg, per os; days 1-28, **AZA:** 75 mg/m^2^, SC or IV; days 1-7/28-day cycle	ITT: 431 pts **AZA-VEN** group: 286 pts **AZA** group **(placebo):** 145 pts Median follow-up: 20.5 mts	**AZA-VEN** group: Median OS: 14.7 mts, CRc: 66.4%, CR: 36.7% **AZA** group **(placebo):** Median OS: 9.6 mts, CRc: 28.3%, CR: 17.9% p<0.001	**AZA-VEN** group: SAEs: 83% Thrombopenia: 45% Neutropenia: 42% Febrile Neutropenia: 42% **AZA** group **(placebo):** SAEs: 73% Thrombopenia: 38% Neutropenia: 28% Febrile Neutropenia: 19%	Completed	NCT02993523
Pratz KW et al, Am J Hematol, 2024 (120)	Phase III, Interventional, Randomized	Long-term follow-up of VIALE-A: Venetoclax and azacitidine in chemotherapy-ineligible untreated acute myeloid leukemia	**VEN:** target dose 400 mg, per os; days 1-28, **AZA:** 75 mg/m^2^, SC or IV; days 1-7/28-day cycle	ITT: 431 pts **AZA-VEN** group: 286 pts, **AZA** group **(placebo):** 145 pts Median follow-up: 43.2 mts	**AZA-VEN** group: Median OS: 14.7 mts, Estimated 24-month OS rate: 37.5% **AZA** group **(placebo):** Median OS: 9.6 mts, p<0.001 Estimated 24-month OS rate: 16.9%	**AZA-VEN** group (any grade): Thrombopenia: 47% Neutropenia: 43% **AZA** group **(placebo)** (any grade): Thrombopenia: 42% Neutropenia: 29% Any grade: similar Gastrointestinal and Hematological: similar	Completed	NCT02993523
Pollyea DA et al, Clin Cancer Res, 2022 (17)	Phase III, Interventional,Randomized Phase Ib, Interventional, Non-randomized	Impact of Venetoclax and Azacitidine in Treatment-Naïve Patients with Acute Myeloid Leukemia and IDH1/2 Mutations	**VEN:** target dose 400 mg, per os; days 1-28, **AZA:** 75 mg/m^2^, SC or IV; days 1-7/28-day cycle	**AZA-VEN** group: 286 pts, **AZA** group **(placebo):** 145 pts VEN+AZA (1 arm)	**AZA-VEN** group (IDH1+IDH2 mut.): Median OS: 24.5 mts, CRc: 79% Median DoR: 29.5 mts **AZA-VEN** group (IDH2 mut.): Median OS: NR CR: 56%, CRc: 86% (IDH1 mut.): Median OS: 15.2 mts CR: 27%, CRc: 66.7% **AZA** group **(placebo)** (IDH1+IDH2 mut.): Median OS: 6.2 mts, CRc: 11% Median DoR: 9.5 mts p<0.001	No unexpected toxicities in the VEN-AZA arm	Completed	NCT02993523 NCT02203773
Roboz GJ et al, Blood, 2020 (21)	Phase I, Interventional, Single Group, Open Label	Study of Orally Administered AG-120 (Ivosidenib) in Subjects With Advanced Hematologic Malignancies With an IDH1 Mutation ———————————— Ivosidenib induces deep durable remissions in patients with newly diagnosed IDH1-mutant acute myeloid leukemia	**IVO:** 500 mg per os/daily; days 1-28/28-day cycle	**IVO** group: 34 pts Median follow-up: 23.5 mts	**IVO** group: Median OS: 12.6 mts, CRc: 42.4%, CR: 30.3% Median DoCRc: NR	Any grade: Diarrhea: 53% Fatigue: 47% Nausea: 38% Decreased appetite: 35% Differentiation syndrome: 18% (grade ≥3: 9%)	Ongoing - Recruiting	NCT02074839
Montesinos P et al, N Engl J Med, 2022 (22)	Phase III, Interventional, Randomized, Double Blind	Study of AG-120 (Ivosidenib) vs. Placebo in Combination With Azacitidine in Participants With Previously Untreated Acute Myeloid Leukemia With an IDH1 Mutation	**IVO:** 500 mg per os/daily; days 1–7 or 1–5 and 8-9/28-day cycle, minimum of 6 cycles **AZA:** 75 mg/m^2^, SC or IV; days 1–7 or 1–5 and 8-9/28-day cycle, minimum of 6 cycles	ITT: 146 pts **IVO-AZA** group: 72 pts, **AZA** group **(placebo):** 74 pts Median follow-up: 12.4 mts	**IVO-AZA** group: Event-free 12 mts: 37% Median OS: 24 mts CRc: 53%, Median DoR: 22.1 mts **AZA** group **(placebo):** Event-free 12 mts: 12% Median OS: 7.9 mts P=0.001 CRc: 18%, Median DoR: 9.2 mts	**IVO-AZA** group: Febrile neutropenia: 28% Neutropenia: 27% Differentiation syndrome (any grade): 14% Infections (any grade): 28% Bleeding (any grade): 41% **AZA** group **(placebo):** Febrile neutropenia: 34% Neutropenia: 16% Differentiation syndrome (any grade): 8% Infections (any grade): 49% Bleeding (any grade): 29%	Completed	NCT03173248
Short NJ et al, J Clin Oncol, 2024 (131)	Phase I, II Interventional, Single Group, Open Label	Azacitidine, Venetoclax, and Gilteritinib in newly diagnosed and relapsed or refractory FLT3-Mutated AML	**VEN:** target dose 400 mg, per os; days 1-28 (1^st^ cycle), days 1-21 (next cycles) **AZA:** 75 mg/m^2^, SC or IV; days 1-7/28-day cycle **Gilteritinib:** 80 or 120 mg (phase I), 80 mg (phase II) per os, once daily; days 1-28	52 pts **ND** AML group: 30 pts Median follow-up: 19.3 mts **R/R** AML group: 22 pts	**ND** AML group: CRc: 96% FLT3-ITD MRD < 5 X 10^-5^ (within 4 cycles): 65% Median RFS, OS: NR 18-month RFS: 71% 18-month OS: 72% **R/R** AML group: CRc: 27%	Infection (62%) Febrile neutropenia (38%) More frequent in the R/R group	Ongoing - Recruiting	NCT04140487
Yilmaz M et al, Blood, 2023 (132)	Phase I, II Interventional, Single Group, Open Label	Quizartinib, Decitabine, and Venetoclax in Treating Participants With Untreated or Relapsed Acute Myeloid Leukemia or High Risk Myelodysplastic Syndrome	**VEN:** target dose 400 mg, per os; days 1-14, days 1-21 (if persistent leukemia)/28-day cycle **DAC:** 20 mg/m^2^, IV; days 1-10/28-day cycle (induction), days 1-5/28-day cycle (next cycles), **Quizartinib:** 30 or 40 mg per os, once daily; days 1-28/28-day cycle	50 pts **ND** AML group: 10 pts Median follow-up: 15 mts **R/R** AML group: 40 pts Median follow-up: 33 mts (gilteritinib-exposed R/R cohort)	**ND** AML group: CRc: 100% MRD MFC 10^-4^: 78% FLT3 PCR MRD 10^-2^ - 10^-3^: 90% Median OS: NR **R/R** AML group: CRc: 68% MRD MFC 10^-4^: 29% FLT3 PCR MRD 10^-2^ - 10^-3^: 36% (gilteritinib-exposed R/R cohort) Median OS: 7.1 mts 1-year OS: 22%	**Quizartinib 30 mg/day: RP2D** **ND** AML group: pneumonia: 40% neutropenic fever: 50% sepsis: 10% bacteremia: 20% **R/R** AML group: pneumonia: 73% neutropenic fever: 53% sepsis: 18% bacteremia: 15%	Ongoing - Recruiting	NCT03661307
Lachowiez CA et al, Blood Cancer Discov, 2023 (133)	Phase Ib, II Interventional, Single Group, Open Label	Ivosidenib and Venetoclax With or Without Azacitidine in Treating Patients With IDH1 Mutated Hematologic Malignancies	Phase Ib (28-day cycles) DL1: IVO 500 mg +VEN 400 mg DL2: IVO 500 mg +VEN 800 mg DL3: IVO 500 mg +VEN 400 mg + AZA 75 mg/m^2^ DL4: IVO 500 mg +VEN 800 mg + AZA 75 mg/m^2^ **VEN:** days 1–14 per os **IVO:** initiation cycle 1, day 15 and then continuously per os **AZA:** days 1–7 SC or IV	**IVO+VEN+AZA** 19 pts (myeloid malignancies) 12 pts (AML) **IVO+VEN** 12 pts (myeloid malignancies) 10 pts (AML) **ALL STUDY PTS** 31 pts (myeloid malignancies) 22 pts (AML) Median follow-up: 24 mts	**IVO+VEN+AZA** CRc: 90% EFS: NR 12-month EFS: 84% OS: NR 24-month OS: 75% MRD (–): 75% **IVO+VEN** CRc: 83% EFS: 11 mts 12-month EFS: 50% OS: 42.1 mts 24-month OS: 58% MRD (–): 50% **ALL STUDY PTS** Median DoR: 34.6 mts Median EFS: 36 mts Median OS: 42 mts	No significant difference observed between IVO+VEN vs IVO+VEN+AZA cohorts Febrile neutropenia: 29% Lung infection: 19% Differentiation syndrome: 10% TLS: 6.5%	Ongoing - Recruiting	NCT03471260
Bataller A et al, Blood, 2024 (134)	Phase II Interventional, Prospective, Single Group, Open Label	Phase II Study of Cladribine with Low dose Cytarabine and Venetoclax alternating with Azacytidine and Venetoclax for Newly Diagnosed Acute Myeloid Leukemia	**Induction:** **CLAD:** 5 mg/m^2^, days 1-5 **LDAC:** 20 mg/m^2^, twice daily, SC, days 1-10 **VEN:** 400 mg, days 1-21 **Consolidation:** 2 courses **VEN+AZA** (75 mg/m^2^, days 1-7) 2 courses **LDAC+VEN+CLAD**(5 mg/m^2^, days 1-3)	141 pts Median follow-up: 28 mts	CRc: 85% MRD (–) MFC: 78% among all CRc pts 2-year OS: 62% (total) EFS: 55% 2-year OS: 75% among all MRD (–) pts 2-year OS: 85% for the transplanted and 55% for non-transplanted	No unexpected toxicities in all pts	Ongoing - Recruiting	NCT03586609

Regarding toxicity, grade ≥3 is provided, unless otherwise stated for any grade side effects. AZA, Azacitidine; CLAD, Cladribine; CR, complete remission; CRc, composite complete remission; DAC, Decitabine; DL, Dose Level; DoR, duration of remission; DoCRc, duration of composite complete remission; EFS, event free survival; FLT3-ITD, FMS-related tyrosine kinase 3-internal tandem duplication; ITT, intention to treat; IV, intravenously; IVO, Ivosidenib; LDAC, low-dose cytarabine; MFC, multicolor flow cytometry; MRD, measurable residual disease; mts, months; mut, mutations; ND, newly diagnosed; NR, not reached; ORR, Overall Response Rate; OS, Overall Survival; PCR, polymerase chain reaction; pts, patients; PFS, Progression free survival; RFS, Relapse free survival; RP2D, recommended phase 2 dose; RR, Response Rate; R/R, Relapsed/Refractory; SAEs, serious adverse events; SC, subcutaneously; TLS, tumor lysis syndrome; VEN, Venetoclax.

The biological basis for the favorable response of the IDH-mutated AML (especially IDH2-mutated AML) to VEN-AZA is largely unknown. It has been documented that the oncometabolite (R)-2-hydroxyglutarate (2-HG) disrupts mitochondrial function via inhibition of cytochrome c oxidase, thereby increasing the efficacy of VEN in IDH-mutated AML (23, 24). The latter is a possible theory explaining the remarkable response of IDH-mutated AML to VEN.

It has also been reported that R140 IDH2 mutations have a more favorable prognosis, compared to R172 IDH2 mutations and R132 IDH1 mutations in AML patients (25–27). However, such an association has not been observed in a recent large cohort evaluating the type and incidence of IDH mutations in AML (28). Intriguingly, R140 IDH2 mutations are highly associated with nucleophosmin-1 (NPM1) mutations (27). NPM1 is mutated in almost one third of AML patients and has been linked with a favorable prognosis in *de novo* AML, but with a dismal prognosis in relapsed or refractory (R/R) AML (28–30). The association of NPM1 with R140 IDH2 might justify the favorable prognosis of R140 IDH2 mutated AML, especially in patients receiving VEN-AZA (18, 19). Moreover, triplet regimens targeting IDH1/IDH2 mutations in combination with VEN/AZA, either at the presence of IDH mutations at diagnosis or at AML relapse, are analyzed in the future directions part of this work.

Furthermore, an NPM1-mutated AML is an excellent target for VEN-based therapies (18, 30). Patients with this leukemic subset receiving low-dose cytarabine (LDAC) plus VEN exhibit a response rate of 78% and a median OS of more than 2 years (31, 32). Furthermore, bone marrow blast reductions >50% might be the case only after 7 days of treatment with VEN in the NPM1-mutated AML (33, 34). Interestingly, a higher benefit group has been observed with a median OS expectation of 39 months for a subset of patients with NPM1-mutated AML lacking either TP53, FLT3-ITD, KRAS or NRAS variants (18, 32). The combinations of VEN plus NPM1 downstream targets, such as menin and exportin 1 (XPO1) inhibitors are described in the future directions part of the present article.

A possible molecular mechanism linking VEN with NPM1-mutated AML derives from studies in the human monocytic leukemia cell line THP-1 harboring the NPM1 mutation. VEN inhibits the anti-apoptotic activity of the transcription factor NF-κB and suppresses BCL2, which selectively sequesters the apoptotic protein BAX, thereby targeting the anti-apoptotic pathways observed in NPM1-mutated AML (35, 36). VEN blocks the ability of the anti-apoptotic protein BCL2 to bind to BAX/BAK, acting as a BH3-mimetic, because of its unique structural similarity to BH3-containing proteins, resulting in competitive inhibition (36, 37).

It has been proposed that the evaluation of BCL2 levels at AML diagnosis by mRNA quantification or immunohistochemistry, could serve as a biomarker predicting the response to VEN or anti-BCL2 treatment, because elevated BCL2 protein levels correlate with higher sensitivities to VEN (38). However, the amount of BCL2 capable of binding and sequestering proapoptotic proteins (BAX, BIM) provides a better explanation for this sensitivity, rather than high BCL2 levels alone (39, 40). Intriguingly, biomarkers predicting response to VEN or anti-apoptotic therapy in AML patients might involve BCL2 mutations of the VEN binding site or other BCL2 family gene mutations (8). Moreover, patients with core binding factor (CBF) AMLs exhibit CR/CRi response rates of 80% (41) or 70% (42) when treated with VEN/HMAs, with a significant better response for patients with inv (16) compared to t (8, 21) patients (100% vs 30% achieving CR/CRi after the first cycle) (39, 42). Clinical trials are testing the latter finding (NCT04628026). Finally, CR rates over 90% with measurable residual disease (MRD) clearance have been reported for the favorable risk subgroup with the combination of VEN plus CLIA (cladribine, idarubicin, cytarabine) or FLAG-Ida (fludarabine, cytarabine, granulocyte colony-stimulating factor, idarubicin) (43).

The ELN risk classifications of 2017 (44) and 2022 (45) failed to stratify prognostic outcomes for frail AML patients ineligible for allogeneic hematopoietic stem cell transplantation (HSCT) receiving non-intensive chemotherapy, such as VEN-AZA (46). These classifications were formed incorporating the intensive treatment protocols applied to AML patients and excluding less-intensive treatments, like VEN-AZA, because the latter therapeutic approaches are more novel comparatively (46).

The molecular signature of the four genes TP53, KRAS, NRAS and FLT3-ITD guides prognosis and classifies AML patients who received VEN-AZA to three different prognostic groups. A favorable risk group with all the aforementioned four genes negative (median OS: 26.5 months), an intermediate risk group by positive FLT3-ITD and/or mutated KRAS and/or mutated NRAS (median OS: 12.1 months) and an adverse risk group with mutated TP53 (median OS: 5.5 months) (46). Moreover, this four-gene prognostic combination was further confirmed by *Bataller* et al. (47). Novel therapeutic avenues are necessary for AML patients harboring KRAS, NRAS and FLT3-ITD mutations (also named activating signaling gene mutations), because they exhibit a relatively unfavorable outcome with VEN-AZA (47). Intriguingly, this evaluation of response to VEN with the contribution of the four abovementioned genes does not include AML patients with a prior myelodysplastic syndrome (MDS) or a prior myeloproliferative neoplasm (MPN) or prior exposure to HMAs before the initiation of VEN and HMAs, due to the exclusion of such patients from the relevant clinical trials of VEN in AML.

Intriguingly, assessment of NPM1 MRD evaluated by quantitative real-time polymerase chain reaction (qRTPCR) provides valuable prognostic intuition in AML patients who received VEN combinations. The achievement of bone marrow (BM) MRD negativity after 4 cycles of combination treatment with VEN and HMAs demonstrated a 2-year OS of 84%, compared with 46% for MRD positive by the end of cycle 4 for AML patients (48). Thus, a patient group with a better outcome has been described for NPM1-mutated AML patients who received VEN. VEN-based combination treatment is a very promising targeted therapy for NPM1-mutated AML, because it has been associated with durable molecular remission and increased OS (18, 48). Even in patients with NPM1 molecular failure, VEN combinations serve either as a bridge to transplant or as definitive therapy, as 71% of these patients become MRD negative (49). MRD monitoring is emerging and plays a complementary role in defining prognosis for AML patients receiving VEN-AZA (50). Thus, AML patients with CRc and MRD < 10^-3^ after treatment with VEN-AZA had longer duration of response, EFS and OS, compared to responding patients with an MRD ≥ 10^-3^ (50).

Another key element for the prognosis and the response to VEN-AZA in NPM1-mutated AML is the co-existence of activating signaling mutations (FLT3-ITD, KRAS, NRAS). NPM1-mutated AML without these mutations has a median OS of 39 months, in contrast to only 9.9 months when these kinase signaling mutations are present in parallel with the NPM1 mutation (51). The results are similar for IDH2 mutated AML, RUNX1 mutated AML and AML with myelodysplasia related (MR) gene mutations, depending on the absence or presence of an activated kinase pathogenic variant (51).

Additionally, patients with DDX41 mutated AML exhibit a particularly favorable outcome with 91.1% of OS probability at 2 years (median OS not reached) after being treated with VEN-AZA (52, 53). Moreover, *Weng G* et al. demonstrated that ASXL1, NPM1 and chromatin-cohesin complex genes predict superior response to VEN (54). Improved responses of AML patients with ASXL1 mutations (55) or with splicing factor gene mutations (56), along with exceptional positive outcomes for those harboring MR gene mutations with the combination of VEN plus HMAs have also been reported (57).

Interestingly, the elimination of a population of leukemic stem cells (LSCs) determines the response to the combination of VEN-AZA. The proteinic expression of BCL2, BCL-xL, and MCL1 in these LSCs, not only significantly predicts the response to the combination with high sensitivity and specificity, but correlates the combinatorial levels of BCL2 family members with an increased EFS in AML patients as well (58). The flow cytometry-based “Mediators of apoptosis combinatorial score” (MAC-Score) incorporates a combination of the expression of the abovementioned BCL2 family members, reliably predicting the response of AML patients to VEN-AZA. LSCs of refractory or relapsed patients to the combination exhibit perturbed apoptotic dependencies (58).

The relevant factors contributing to a favorable response to VEN-AZA in AML patients are shown in the upper part of **Table 2**.

**Table 2:** Factors predicting a favorable response (upper part of the table) and factors causing resistance (lower part of the table) to the combination of VEN-AZA in AML patients.

Factors predicting a favorable response to the combination of VEN-AZA in AML patients	
Factor	Rational
IDH1 or IDH2 mutations	OS: 24.5 months, CRc: 79% Disruption of mitochondrial function by 2-HG via inhibition of cytochrome C oxidase
IDH2 mutations IDH2 mutations on the absence of activated kinase pathogenic variant [KRAS (–), NRAS (–), FLT3-ITD (-)]	OS: NR, CRc: 86% Longer median OS
IDH1 mutations	OS: 15 months, CRc: 67%
R140 IDH2 mutations	Highly associated with NPM1 mutations
NPM1-mutated AML MRD (-) after 4 cycles of VEN-AZA NPM1-mutated AML and KRAS (-), NRAS (-), FLT3-ITD (-)	2-year OS: 84% Median OS: 39 months
TP53 (-), KRAS (-), NRAS (-), FLT3-ITD (-)	Median OS: 26.5 months
AML patients with CRc and MRD < 10^-3^ after VEN-AZA	Longer duration of response, EFS and OS
RUNX1-mutated AML on the absence of activated kinase pathogenic variant [KRAS (-), NRAS (-), FLT3-ITD (-)]	Longer median OS
AML with myelodysplasia related (MR) gene mutations on the absence of activated kinase pathogenic variant [KRAS (-), NRAS (-), FLT3-ITD (-)]	Longer median OS
DDX41-mutated AML (particular favorable outcome)	Median OS: NR, 2-year OS: 91.1%
ASXL1, chromatin-cohesin complex genes, splicing factor gene mutations	Superior response
The elimination of a population of leukemic stem cells with high proteinic expression of BCL2, BCL-xL, MCL1	The combinatorial levels of BCL2 family members: increased EFS
Factors causing resistance to the combination of VEN-AZA in AML patients	
Factor	Rational
VEN monotherapy	Intrinsic resistance of leukemic cells to VEN
VEN-AZA: 50% relapse after an initial response	Acquired resistance to the drug
TP53, KRAS, NRAS, FLT3-ITD mutations	Mediators of VEN resistance in AML
Biallelic TP53 abnormalities or TP53 loss	VEN resistance, delayed BAX activation, increase of BAX apoptotic threshold
RAS-mutant LSCs	Exhibit deranged BCL2 family protein gene expression, relapse with monocytic characteristics with a distinct MCL1-dependent transcriptomic profile
RAS/MAPK/MCL1 pathway	Methylation and metabolomic single cell analysis
Increased MCL1 expression (the hallmark of intrinsic VEN resistance) and expansion of leukemic cells	Mice harboring cells maintaining MCL1 expression acquire AML relapse and VEN resistance after initial response
BCL-xL highly expressed in erythroid/megakaryocytic AML	VEN resistance
MCL1 and BCL-xL inhibit BAX expression	Failure in apoptosis induction in MARCH5 AML cells and VEN resistance
Various types of BAX mutations	VEN resistance
Mitophagy through the overexpressed modulator MFN2	VEN resistance in AML
Unique metabolic profiles, increased levels of CTP, dCTP, modifications in fatty acid and amino acid metabolism, elevated levels of CD36, increased activity of glycolysis	VEN-AZA resistant AML cell lines
Upregulation of nicotinamide pathway	VEN resistance in AML, deranged metabolic pathways in AML patients receiving VEN

## Resistance to venetoclax

Even though VEN targets BCL2 overexpression in AML patients, the monotherapy of the drug has minimal therapeutic properties, hinting a mechanism of intrinsic resistance of the leukemic cells to the drug (59). Moreover, almost 27% of AML patients show primary resistance and more than 50% relapse after an initial response to VEN-AZA, suggesting the existence of acquired resistance, in parallel with the intrinsic one (2). Nevertheless, the precise molecular mechanisms driving resistance to VEN in AML patients have yet to be identified.

The molecular signature of the four genes TP53, KRAS, NRAS and FLT3-ITD not only defines response to VEN-AZA, but also determines resistance to VEN (60, 61). In other words, TP53, KRAS, NRAS and FLT3-ITD are mediators of VEN resistance in AML (5, 60, 62, 63). Adaptive resistance is often associated with biallelic TP53 abnormalities or TP53 loss or kinase activation, in particular FLT3 ITD or RAS mutations (18, 60, 62). Interestingly, RAS mutations are late events in leukemogenesis and can originate from a different clone, compared to the ancestral ones. RAS-mutant leukemic stem cells (LSCs) are resistant to VEN, exhibit deranged BCL2 family proteins gene expression and relapse with monocytic characteristics (64, 65). Resistant monocytic AML exhibits a distinct transcriptomic profile and is MCL1-dependent to maintain survival (65, 66). DNA sequencing experiments also provided evidence of selection of RAS-mutated clones in AML patients treated with VEN, conferring resistance to the drug (65, 67). This resistance and AML relapse is mainly driven by the RAS-mutant leukemic stem cells (LSCs) alone, rather than the monocytic differentiation state of the LSCs, which is the result of the initially subclonal, oncogenic RAS driver variants after selective clonal pressure, caused by VEN treatment (64). Finally, the activation of RAS/MAPK/MCL1 pathway has been recognized as a main mechanism of VEN resistance in AML through gene and protein expression, along with methylation and metabolomic single cell analysis (67).

More precisely, AML patients receiving VEN-AZA, might harbor FLT3-ITD mutation at diagnosis, or may develop the same mutation at relapse, demonstrating secondary or acquired VEN resistance during the course of the disease (68). Moreover, VEN resistance can be observed in AML patients, through activation of intracellular signaling pathways by mutations in genes involving key kinases, such as RAS or PTPN11 (68, 69). FLT3-ITD triggers PI3K-protein kinase B (Akt), RAS-MAPK and STAT5 pathways. STAT5 controls BCL-xL and Akt controlling MCL1 stabilization in FLT3-ITD leukemic cells (70). The presence of FLT3-ITD mutations either at diagnosis or driving AML relapse after VEN-AZA treatment, exhibits apparent therapeutic applications with triplet regimens targeting FLT3-ITD, highlighted in the future directions part of this work.

Defective TP53 has a major role in determining VEN resistance in AML, because TP53 knockout impairs apoptotic cell death caused by VEN (71). An initial activity of small degree with a rapid evolution and growth of subsequent TP53-mutant clones causing a quick relapse has been observed in TP53-mutated AML (18). Suboptimal concentrations of VEN cause delayed BAX activation and the latter increases BAX apoptotic threshold (72). In this regard, it is more difficult for VEN to induce apoptosis of leukemic cells, due to the elevated apoptotic threshold. TP53 knockout delays activation of the apoptotic proteins BAX and BAK and thus apoptosis, because the target genes of TP53, such as NOXA, BIM and PUMA are implicated in BAX and BAK activation (62, 72). Overall, VEN is not effective in TP53-mutated AML, as defective TP53 confers resistance to the drug (73).

Because VEN is a selective inhibitor of BCL2 protein expressed in AML, other anti-apoptotic BCL2 proteins, such as BCL-xL (74), MCL1 (66, 67, 75), BCL2A1 (76, 77) and BFL1 serve as mediators for primary or acquired secondary resistance to VEN by leukemic cells, via alterations of the apoptotic pathways (5–7, 69). These anti-apoptotic proteins through specific interactions cause sequestration of BH3-only proteins, preventing them from synergizing with BAK or BAX proteins to initiate apoptosis (78). BAX inactivation, along with TP53, leads to VEN resistance in AML (71). It has been observed that AML relapse arises in mice harboring cells that maintain MCL1 expression, after an initial successful inhibition of MCL1 and impermanent eradication of leukemic cells (79). Additionally, MCL1 is the highest expressed BCL2 family protein in AML, thereby proving the relation between MCL1 and the progression of the disease, along with the expansion of leukemic cells (60, 80–84). Selective BCL2 inhibition by VEN leads to the death of leukemic cells. Nevertheless, this BCL2 inhibition is antagonized via an increase of MCL1 expression by AML, which is the hallmark of intrinsic VEN resistance (38, 67, 85). It has been observed that genetic silencing of MCL1 reverses the resistance to VEN and restores the anti-leukemic properties of the drug (38, 67). In addition, BCL-xL is highly expressed in erythroid/megakaryocytic AML and it is responsible for VEN resistance in this leukemic subset (74).

As VEN is directly attached to the BCL2 BH3-binding grooves of the anti-apoptotic proteins (38, 86), the protein BAX is released from BCL2 and upon its self-assembly, novel pores are constructed, permeabilizing the outer membrane of the mitochondria, which becomes vulnerable to external and internal stress factors (87, 88). The subsequent release of cytochrome c from the mitochondria, induces cellular apoptosis, via activation of the caspase programmed cell death pathway (87, 88).

Intriguingly, mutations in the BCL2 family members after initial response to VEN treatment is another major mechanism driving VEN resistance in AML. Resistance-associated point mutations in the BCL2 protein, such as BCL2 Asp103Glu, Val148Leu and Phe104Leu have recently been identified in AML (89). These BCL2 variants are polyclonal, arise during VEN therapy and reduce the affinity of BCL2 with the drug, establishing secondary resistance to VEN in AML, despite VEN dose escalation (81, 89). Moreover, secondary inactivating frameshift/nonsense or missense mutations involving the BAX apoptotic gene have also been described in relapsed AML patients who received VEN (90).

It has been documented that the MARCH5 mitochondrial E3 ligase complex, which consists of MARCH5, UBE2K and UBE2J2 controls VEN resistance and sensitivity, through modulation of BAX saturation of the anti-apoptotic proteins (91). When genome-wide CRISPR/Cas9 techniques were applied in a resistant to VEN AML mouse model, the levels of MARCH5, UBE2K and UBE2J2 were significantly decreased after exposure to VEN, suggesting a combined lethal effect. However, after VEN treatment, MCL1 and BCL-xL entrapped BAX, which leads to failure in the induction of apoptosis in MARCH5 AML cells (91). Furthermore, BAX knockdown resulted in VEN resistance (75). Nevertheless, in MARCH5 knockout cells, the released BAX from BCL2, failed to attach to MCL1, because another molecule (NOXA) occupied the respective MCL1 BH3-binding domains. In this way, BAX effectively caused mitochondrial apoptosis and overcame the MCL1-induced resistance to VEN in AML (91). Intriguingly, the leading role of MCL1 in the development of resistance to VEN treatment in AML has also been confirmed in the aforementioned experiments. Finally, various types of BAX mutations, leading to different alterations in the structure and the function of the BAX protein, have been established in relapsed AML patients who received VEN (90).

Another interesting mechanism involving the mitochondrial axis and promoting resistance to VEN in AML is mitophagy, which is a selective type of autophagy in which mitochondria are destructed, through autophagic degradation (92, 93). MFN2, a mitophagy modulator, is responsible for inducing resistance in BH3 mimetics, like VEN in AML (93). Thus, MFN2 overexpression leads to increased interactions between mitochondria and endoplasmic reticulum, causing enhanced mitochondrial clearance and resistance to VEN, which in the abovementioned cell background of mitophagy, cannot function as an effective drug in AML (93). Furthermore, a gene implicated in mitochondrial metabolism is CLPB. This gene interacts with the OPA1 protein, maintains mitochondrial cristae structure and it is overexpressed upon VEN resistance in AML (94).

Unique VEN-AZA resistant AML cell lines have been developed showing more than 300-fold persistent resistance compared to the parental lines. These cells have unique metabolic profiles, increased levels of cytidine triphosphate (CTP) and deoxycytidine triphosphate (dCTP), modifications in fatty acid and amino acid metabolism, elevated levels of the fatty caid transporter CD36, increased levels of MCL1, decreased levels of BAX and increased utilization and reliance on glycolysis (75). Intriguingly, pharmaceutical inhibition of glycolysis re-sensitized the resistant cells to VEN-AZA (75). AML cells generate the energy needed for their metabolic functions from the citric acid cycle and the oxidative phosphorylation (OXPHOS). VEN inhibits the electron transport chain complexes I, II and IV, thereby decreasing OXPHOS and destroying leukemic cells (84, 94). However, AML cells develop resistance to VEN, because they upregulate the nicotinamide pathway (95) or the fatty acid metabolism (96).

Another biological mechanism driving VEN resistance in AML involves ATP-binding cassette (ABC) transporters, which are major regulators of drug efflux. They are implicated in detoxification, cell signaling and metabolism by transporting substrates, like metabolites, nucleosides, ions, hormones, lipids and cytotoxic drugs across the cell membrane (97). ABC transporters, such as ABCC1, have the potential to efflux anticancer agents, as their overexpression has been associated with multidrug resistance and poor response to chemotherapy (98). It has been demonstrated that ABCC1 overexpression induces resistance to VEN in AML by reducing the intracellular drug levels, thereby predicting poor response to VEN (99). Conversely, ABCC1-specific export of glutathionylated substrates leading to the inhibition of glutathione metabolism, increases the response to VEN in AML patients. In other words, glutathione metabolism and ABCC1 limit VEN efficacy in AML (99). The molecular mechanisms describing the deranged metabolic dependency in AML patients receiving VEN have been extensively reported and are beyond the scope of this review (81, 82, 84, 100, 101).

Finally, the immune microenvironment of AML causes increased production of pro-inflammatory cytokines, driving leukemic progression (102). In particular, aberrant myeloid cell proliferation of monocytic origin, observed in del7/7q AML patients, leads to enhanced IFNγ signaling, which is a key feature of VEN resistance in AML (103).

The corresponding mechanisms causing resistance to VEN-AZA in AML patients are described in the lower part of **Table 2**.

## Discussion - concluding remarks – future directions

Therapeutic ways of overcoming VEN resistance in AML target MCL1 and BCL-xL (5, 60–62). There are direct MCL1 inhibitors, like S63845/S64315 (MIK665) (104), AZD5991 (105), AMG-176 (tapotoclax) (106) and AMG-397 (murizatoclax) (107) or indirect MCL1 inhibitors, such as CDK9 inhibitors [alvosidib (108, 109), dinaciclib (110), AZD4573 (111, 112), PIK-75 (113)], deubiquitinase inhibitors (114) and ceramide (SPHK1 inhibitor) (115, 116).

However, the results of direct MCL1 inhibitors in clinical trials were not satisfactory, due to side effects, like myelosuppression (104, 106, 117) or cardiotoxicity (107). Importantly, MCL1 knockout mice show fatal cardiac failure (118, 119). Conversely, heterozygous mice for MCL1 exhibit no cardiac manifestations, thereby rendering decreased dosing a possible effective future therapeutic strategy for direct MCL1 inhibitors in AML. Alternatively, indirect MCL1 inhibitors have a better tolerability with less toxicities and are under extensive research as potent drugs overcoming VEN resistance in AML (112). Moreover, targeting BCL-xL is a potential treatment option in erythroid/megakaryoblastic leukemias resistant to VEN (74).

An updated safety analysis regarding long-term use of VEN-AZA in elderly AML patients or those with comorbidities identified no novel safety concerns. Treatment emergent adverse events (TEAEs) were similar between the VEN-AZA arm versus the placebo-AZA arm. Slightly higher rates of hematologic TEAEs were reported in the VEN-AZA arm, compared to the placebo-AZA arm. These were grade ≥3 thrombocytopenia (46% vs 40%), neutropenia (43% vs 29%) and febrile neutropenia (43% vs 19%) (120). With a median follow-up of 43.2 months, 86% of the VEN-AZA arm and 77% of the control arm had serious TEAEs. Fatal TEAEs reached 25% in the VEN-AZA arm, compared to 22% in the placebo-AZA arm (120). Since VEN-AZA is applied for a prolonged use or in combination with other drugs/therapies in elderly AML patients, a retrospective study evaluated the possible cardiac complications. Cardiomyopathy, pericarditis/effusions, along with non-ST elevation myocardial infarction were the major cardiac complications observed in the 7.6% of a cohort of newly diagnosed (ND) AML patients. Males were more likely and DNMT3A-mutated patients less likely to be affected. These cardiac complications were associated with a trend towards shorter OS and CRc (121). Finally, the general guidelines for preventing tumor lysis syndrome (TLS), along with the dose modifications for VEN with concomitant use of CYP3A (protection from fungal infections) and P-gp inhibitors are well known and applied from the clinicians (122).

Potential strategies to overcome resistance to VEN-AZA in AML include combination with other agents like FLT3, IDH, RAS, menin, XPO1 and immune checkpoint inhibitors. Current triplet regimens under clinical trials involving VEN/HMAs with FLT3 inhibitors are either with quizartinib (NCT03661307, NCT04687761) or gilteritinib [NCT04140487 (**Table 1**), NCT05010122, NCT05520567, NCT06317649, NCT06696183] in the frontline setting or in the R/R disease. Gilteritinib is tested in most trials with VEN/AZA and in one trial with VEN plus ASTX727 [oral decitabine (DAC) and the cytidine deaminase inhibitor cedazuridine], whereas quizartinib with DAC plus VEN (NCT03661307, **Table 1**) or either with VEN/AZA or with low-dose cytarabine (LDAC)/VEN (NCT04687761, VEN-A-QUI trial).

Moreover, triplet regimens with IDH inhibitors under clinical trials are the combinations of IVO plus VEN/AZA or VEN in the R/R setting (NCT03471260, **Table 1**) and the combination of ASTX727 plus VEN plus any type of IDH inhibitor (NCT04774393). Another trial involves the combinations of IVO plus VEN (NCT06611839) both in the first line and in R/R AML. Furthermore, the addition of VEN to enasidenib and AZA (NCT03683433) has been associated with 100% CRc in elderly patients with IDH2-mutant AML in the frontline setting (123). Finally, olutasidenib with VEN/AZA (NCT06782542) is under evaluation in IDH1-mutated ND AML patients eligible for intensive induction chemotherapy (phase II OLUVENAZA trial). Another phase Ib/II clinical trial is under conduction testing olutasidenib plus VEN/DAC (NCT06445959) in ND and R/R AML patients.

Menin inhibitors are also under clinical trials to treat ND or R/R KMT2Ar, NUP98r or NPM1c AML. The therapeutic combinations applied in clinical trials involve the triplets of Revumenib plus VEN plus ASTX727 (NCT05360160), Revumenib plus VEN/AZA (NCT06652438), Ziftomenib plus VEN/AZA (NCT05735184, KOMET-007 study) (124) or Bleximenib plus VEN/AZA (NCT05453903). The promising results of these combinations in specific leukemic subsets are being evaluated. Based on the results of the AUGMENT-101 clinical trial (NCT04065399), Revumenib was approved in the United States for R/R acute leukemia with a KMT2A translocation in adult and pediatric patients of 1 year and older (125).

Clinical trials with XPO1 inhibitors, like selinexor or eltanexor in combination with VEN or with VEN/AZA have been completed or are under conduction, either in frontline AML or in the R/R setting. The most important are the combinations of selinexor plus VEN (NCT03955783), selinexor plus VEN/AZA (NCT05736965) or eltanexor plus VEN (NCT06399640). The initial encouraging results are important, and a thorough analysis is underway to observe the efficacy and safety of the abovementioned therapeutic combinations in AML.

Because RAS mutations emerge as a major mechanism driving VEN resistance in AML (64, 81, 101, 126), RAS inhibitors have also been tested. RAS mutations exert their leukemogenic actions through activation of the PI3K, Akt, mTOR, RAF, MEK and ERK downstream signaling pathways (126). Trametinib, a MEK inhibitor, in combination with VEN/AZA showed modest activity and substantial toxicity in R/R AML (NCT04487106), with similar responses to trametinib monotherapy (127). Another MEK inhibitor, cobimetinib, acting through indirect RAS inhibition, along with VEN, showed limited preliminary efficacy similar to VEN monotherapy with significant toxicity in the R/R setting (NCT02670044) (128). However, the combination of VEN with the murine double minute 2 (MDM2) inhibitor idasanutlin exhibited manageable safety and encouraging efficacy in unfit patients with R/R AML (NCT02670044) (129). Future clinical trials targeting RAS and MEK pathways in R/R AML with improved efficacy and acceptable toxicities are essential.

Immune checkpoint (PD1) inhibitors in combination with VEN/HMAs are being tested in R/R AML after initial VEN resistance. Pembrolizumab plus DAC ± VEN (NCT03969446), and Tislelizumab plus VEN/AZA (NCT06536959) are promising future therapeutic targets. Moreover, an ongoing open-label phase I/II study of Relatlimab (anti-LAG-3) plus Nivolumab (anti-PD1) in combination with AZA ± VEN for the treatment of patients with R/R or ND AML has demonstrated initial promising efficacy and manageable toxicity (130). Other agents currently tested with VEN/AZA as triplet regimens to overcome VEN resistance in AML are tagraxofusp, which targets CD123 (NCT05442216), the cyclin-dependent kinase 9 (CDK9) inhibitor QHRD107 (NCT06532058), the histone deacetylase (HDAC) inhibitor chidamide (NCT05305859) and the MDM2 inhibitor siremadlin (NCT05155709).

As more knowledge is established regarding the molecular pathways implicated in VEN resistance in AML patients, more targeted approaches will be applied and novel therapeutic avenues will be discovered. Extensive research is essential in order to decipher the aforementioned complex mechanisms of VEN response and mainly, resistance in AML.
